# Collagen Formulation in Xenogeneic Bone Substitutes Influences Cellular Responses in Periodontal Regeneration: An In Vitro Study

**DOI:** 10.3390/biomimetics10090608

**Published:** 2025-09-10

**Authors:** Priscilla Pelaez-Cruz, Pia López Jornet, Eduardo Pons-Fuster

**Affiliations:** 1 Department of Oral Medicine, Spain Biomedical Research Institute of Murcia (IMIB), University of Murcia, Campus Mare Nostrum, 30120 Murcia, Spain; pv.pelaezcruz@um.es; 2 Human Anatomy Department, IMIB Biomedical Research Institute of Murcia (IMIB), University of Murcia, Campus Mare Nostrum, 30120 Murcia, Spain; eduardo.p.f@um.es

**Keywords:** bone regeneration, collagen, human dental pulp stem cells, periodontal, gene expression

## Abstract

Background: Bone regeneration is a key therapeutic objective in periodontology, particularly in the treatment of alveolar defects caused by periodontal disease, dentoalveolar trauma, or surgical interventions. Among current regenerative strategies, collagen-enriched biomaterials have demonstrated an active role in modulating cellular behavior during bone repair. However, the specific effects of different collagen formulations on human dental pulp stem cells (hDPSCs) have not yet been fully characterized. Objective: To evaluate the impact of xenogeneic bone grafts with and without collagen—OsteoBiol^®^ Gen-Os^®^ (GO), OsteoBiol^®^ GTO^®^ (GTO), and Geistlich Bio-Oss^®^ (BO)—on cell viability, adhesion, migration, osteogenic differentiation, and mineralization potential of hDPSCs, and to explore the molecular mechanisms underlying their effects. Methods: In vitro assays were conducted to assess viability (MTT and fluorescence staining), adhesion (SEM), migration (wound healing assay), and mineralization (Alizarin Red S staining). Gene expression analyses (RT-qPCR) were performed for adhesion/migration markers (*FN*, *SDF-1*, *COL1A1*), angiogenic/proliferation markers (*VEGF*, *FGF2*), and osteogenic differentiation markers (*RUNX2*, *ALP*, *COL1A1*). Results: GO showed a higher early expression of genes associated with adhesion, migration, angiogenesis (*FN*, *SDF-1*, *VEGF* and *FGF2*: *p* < 0.05; *COL1A1*: *p* < 0.01), and osteogenic differentiation (7 days: *COL1A1* and *ALP* (*p* < 0.001)); (14 days: *RUNX2*, *ALP*: *p* < 0.001; *COL1A1*: *p* < 0.05), indicating a sequential activation of molecular pathways and mineralization capacity comparable to the control group. GTO demonstrated the best biocompatibility, with significantly higher cell viability (*p* < 0.05), strong adhesion, and markedly increased mineralization at 21 days (*p* < 0.001), despite moderate early gene expression. BO showed reduced cell viability at 10 mg/mL (*p* < 0.05) and 20 mg/mL (*p* < 0.001), with mineralization levels similar to the control group. Conclusion: Collagen-based xenografts demonstrate favorable interactions with hDPSCs, enhancing viability and promoting osteogenic differentiation. Our findings suggest that beyond the presence of collagen, the specific formulation of these biomaterials may modulate their biological performance, highlighting the importance of material design in optimizing regenerative outcomes. Clinical Significance: The formulation of collagen in xenogeneic bone substitutes may be a determining factor in enhancing periodontal regenerative outcomes by modulating the early cellular response and osteogenic activity in stem cell-based tissue engineering.

## 1. Introduction

Bone regeneration represents a persistent challenge in dentistry and regenerative medicine, particularly when it involves restoring bone structures that have been lost due to disease, trauma, or surgical intervention [1]. In the context of periodontal regeneration, the restoration of alveolar bone lost due to periodontitis remains a significant clinical challenge. The development and optimization of biomaterials capable of supporting periodontal tissue regeneration is therefore of paramount importance, as these materials must not only promote new bone formation but also integrate seamlessly with the complex periodontal environment [2,3].

In this regard, the use of biomaterials has emerged as a viable alternative to autologous grafts. Among these, xenogeneic bone substitutes are widely used due to their availability, biocompatibility, and osteoconductive properties [2].

It is well known that biomaterials used in bone regeneration must be able to promote osteogenesis by acting as a three-dimensional matrix that supports key cellular functions such as cell adhesion, migration, proliferation, and differentiation [4,5]. Collagen type I is the predominant structural protein in the extracellular matrix (ECM) of bone and plays a central role in bone regeneration. In addition to its structural integrity, collagen type I exhibits several advantageous properties, including biodegradability, flexibility, tensile strength, and low immunogenicity. These characteristics position it as one of the most widely utilized polymers in the domain of bone regeneration [6]. It has been documented that collagen, when incorporated into bone graft biomaterials, can modulate alveolar bone resorption during the bone regeneration process [7,8,9]. Additionally, research has shown that collagen can enhance neovascularization by supporting endothelial cell adhesion and stimulating the formation of new blood vessels [10,11].

OsteoBiol^®^ GTO^®^ is a pre-hydrated collagenated heterologous material composed of 80% collagenated cortico-cancellous granules combined with 20% OsteoBiol^®^ TSV gel, which contains collagen type I and III, a thermosensitive synthetic copolymer, and polyunsaturated fatty acids. A recent study has reported that this biomaterial was able to increase Human Mesenchymal Stem Cells recruitment and ALP activity compared to OsteoBiol^®^ Gen-Os^®^, a xenograft with collagen preserved in granules, widely used for its ease of handling and favorable tissue integration, and to Geistlich Bio-Oss^®^, an anorganic bovine xenograft lacking collagen, known for its biocompatibility and long-term volumetric stability [7,12,13,14].

These findings provide evidence that, while the biological role of collagen in tissue regeneration is well established, the influence of its different formulations on cellular function and molecular mechanisms, especially in relation to dental pulp-derived mesenchymal cells, is still limited. Human dental pulp stem cells (hDPSCs) have emerged as a highly promising source of multipotent stem cells due to their unique biological properties [15,16]. Isolated from the soft connective tissue within extracted teeth, hDPSCs exhibit a robust proliferative capacity and a pronounced ability to differentiate, particularly into osteogenic lineages, making them attractive candidates for bone tissue engineering [17,18]. A key advantage is their minimally invasive and easily accessible method of isolation; even a small amount of pulp tissue can yield a substantial number of viable cell colonies [19]. Moreover, hDPSCs display a low immunogenicity and favorable cellular characteristics, further supporting their relevance as a model for regenerative medicine and tissue repair research [20]. Therefore, the present investigation aims to evaluate the effect of three biomaterials with different collagen formulations, OsteoBiol^®^ GTO^®^ (with collagen types I and III in granules and gel), OsteoBiol^®^ Gen-Os^®^ (with collagen type I in granules), and the anorganic biomaterial lacking collagen Geistlich Bio-Oss^®^ (BO), on key processes of bone regeneration, including adhesion, migration, mineralization, and the molecular response in dental pulp mesenchymal cell cultures.

## 2. Materials and Methods

### 2.1. Cell Isolation and Culture

We obtained hDPSCs from third molars extracted from two healthy volunteers aged between 18 and 40 years old. The study was approved by the ethics committee (ID: 631/2024), and all participants provided their written informed consent. Several vials of cells were frozen as a reserve prior to testing. Cell culture passages 3–6 were used in the experiments.

Dental pulp was cut into small pieces with a scalpel and subsequently disaggregated in a 3 mg/mL Collagenase type I and 4 mg/mL Dispase II (Sigma-Aldrich, Merck KGaA, Darmstadt, Germany) solution for 1 h at 37 °C. The digested extracts were strained (through a 70-μm filter) and cultured on a 100 mm cell culture plate. Cell cultures were expanded in complete cell culture medium (alpha-MEM medium supplemented with 10% FBS, 400 mM of penicillin/streptomycin, and 2.5 μg/mL of amphotericin B), and incubated at 37 °C in a 95% oxygen and 5% carbon dioxide mixture.

To induce cell differentiation for mineralization assays, the cells were cultured in complete medium supplemented with 50 μL/mL ascorbic acid, 7.5 mM β-glycerol phosphate, and 1 μM dexamethasone (osteogenic medium).

### 2.2. Characterization of hDPSCs

The mesenchymal phenotype of the isolated cells was confirmed by analyzing the expression of mesenchymal markers using flow cytometry using the Human Mesenchymal Stem Cell Multi-Color Flow Cytometry Kit (R&D Systems, Minneapolis, MN, USA), following the manufacturer’s protocol.

**Figure 1:**
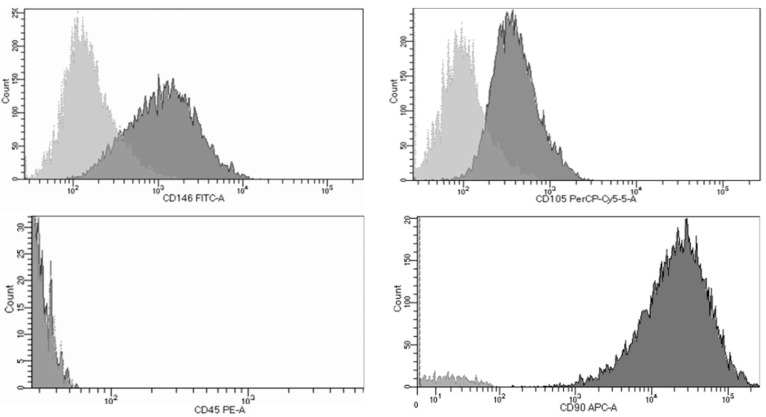
Characterization of human dental pulp stem cells.

More than 95% of the cells were positive for the mesenchymal markers CD105, CD46, and CD90, and negative for CD45 (Figure 1).

### 2.3. Preparation of Bone Substitutes

The three biomaterials were evaluated in viability, migration, gene expression, and mineralization assays in the form of elution. The necessary quantities were weighed on a precision balance in a laminar flow hood, then incubated in a complete medium at 37 °C for 24 h at 37 °C in a 95% oxygen and 5% carbon dioxide mixture. Then, the suspensions were filtered through a 0.22 μm filter to remove solid particles and obtain a conditioned medium free of granules. Additionally, for the scanning electron microscopy assay, the biomaterials were used in direct contact with the cells. The manufacturers’ information is detailed in Table 1.

### 2.4. Evaluation of hDPSC Viability/Proliferation-MTT Assay

hDPSCs were seeded at a density of 5000 cells/cm^2^ in 96-well plates with 200 μL of complete culture medium. After 24 h of incubation, the cells were treated with GO, GTO, and BO at varying concentrations (5, 10, and 20 mg/mL), to ascertain the most efficacious working dose. After the incubation period of 72 h, the medium was replaced with a 1 mg/mL solution of 3-(4,5-dimethylthiazol-2-yl)-2,5-diphenyltetrazolium bromide (MTT), and cells were incubated for 4 h. Then, MTT was removed from the well, and formazan, a product of cell metabolism, was dissolved in 100 μL of dimethyl sulfoxide. Lastly, absorbance was measured with a spectrophotometer (wavelength of 570 nm and a reference wavelength of 690 nm). All the experiments were performed in triplicate. It was determined that a concentration of 5 mg/mL was optimal, and this concentration was employed in all subsequent assays.

### 2.5. Evaluation of hDPSCs Viability-Cell Staining Assay

hDPSCs were cultured as for the MTT assay (in 96-well plates, seeded at 5000 cells/cm^2^, and with 200 μL of complete culture medium). After 24 h of incubation, cells were treated with GO, GTO, and BO. The results were assessed at 72 h using a Viability/Cytotoxicity Assay Kit for Animal Live and Dead Cells (Biotium, Fremont, CA, USA), according to the instructions from the manufacturer. After the incubation period, cells were rinsed with PBS to remove serum esterase activity, and the calcein AM and ethidium homodimer III (Eth-DII) solutions contained in the kit were added. After being held for 30 min at room temperature, the stains were replaced by PBS, and cells were observed using an inverted fluorescence microscope (Zeiss Axio Observer 7, Carl Zeiss AG, Oberkochen, Germany), and photographs were taken of all the wells using an inverted microscope (Nikon Eclipse TE2000-U, Nikon Corporation, Tokyo, Japan).

### 2.6. Evaluation of In Vitro Cell Adhesion and Morphology by Scanning Electron Microscopy (SEM)

The cells were cultured in 24-well plates at a density of 5000 cells/cm^2^ in 1 mL of complete medium. Following 24 h of incubation, cells were treated with GO, GTO, and BO. The biomaterials were added in particulate form, and the cells remained in culture for 72 h. Then, the samples were coated with platinum and examined in a field emission scanning electron microscope (FE-SEM) (Apreo C, Thermo Fisher Scientific, Waltham, MA, USA) at ×100, ×500, and ×1000 magnifications (voltage 5 kV, operating distance 10 mm).

### 2.7. Evaluation of In Vitro Cell Migration-Wound Heal Assay

hDPSCs were cultured in 12-well plates (1 × 10^5^ cells in 2 mL of complete culture medium) and incubated until monolayer confluence. Next, the cells were incubated for 12 h in complete medium with 1% FBS before some areas of the cell monolayer were “wounded” (scraped away) with the sterile tip of a micropipette. After wounding, the medium was replaced (complete medium with 1% FBS), and the cultures were treated with GO, GTO, and BO. Cell migration was quantified by capturing images of the wounded areas at 0 and 24 h of incubation using an inverted microscope with a digital camera (Nikon Eclipse TE2000-U, Nikon Corporation, Tokyo, Japan). The distance between the edges of the damaged area in the cell monolayer was measured by pixel counting using the ImageJ 1.54k digital image processing software (National Institutes of Health [NIH], Bethesda, MD, USA). Migration distance was calculated using the following equation: migration distance = cell-free initial distance − cell-free distance after 24 h. All experiments were conducted in triplicate.

### 2.8. Quantitative Real-Time Reverse-Transcriptase Polymerase Chain Reaction (qRT-PCR)

To evaluate the impact of the biomaterials (GO, GTO, and BO) on the expression of genes implicated in key biological processes, such as cell adhesion, collective migration, proliferation, and angiogenesis in hDPSCs, total RNA was extracted from wound healing assay cells after 24 h of culture using the PureLink™ RNA Micro Kit (Thermo Fisher Scientific, Waltham, MA, USA). Subsequently, the High-Capacity cDNA Reverse Transcription Kit (Thermo Fisher Scientific, Waltham, MA, USA) was used to synthesize cDNA from 2 µg of total RNA.

In addition, to quantify the expression of genes associated with osteogenic differentiation, hDPSCs were cultured in 12-well plates at 5000 cells/cm^2^ with 2 mL of complete culture medium, and then incubated for 7 and 14 days with GO, GTO, and BO in osteogenic medium. All experiments were replicated three times. Subsequently, total RNA was extracted and synthesized, as well as for the quantification of adhesion and migration gene expression, as previously described.

RT-qPCR analyses were performed using a QuantStudioTM 5 Flex Real-Time PCR System (Applied Biosystems, Thermo Fisher Scientific, Waltham, MA, USA) with the PowerTrack™ SYBR™ Green Master Mix (Thermo Fisher Scientific, Waltham, MA, USA). The relative quantity or fold change in the target gene was then normalized to the level of glyceraldehyde-3-phosphate dehydrogenase (GAPDH) and to a control group (untreated cells cultured in complete culture medium for adhesion and migration assays and in osteogenic medium for osteogenic differentiation assays).

The primer sequences for the adhesion and migration genes, the osteogenic differentiation genes, and the endogenous gene are listed in Table 2.

### 2.9. Evaluation of In Vitro Biomineralization-Alizarin Red S Staining Assay

hDPSCs were cultured in 24-well plates seeded at 5000 cells/cm^2^ with a 1 mL of complete culture medium and incubated for 3 days. Next, the medium was replaced by an osteogenic medium, and cells were treated with GO, GTO, and BO. The medium was replaced every 3 days throughout the experimental study (21 days). At the end of the culture period, the cells were washed with PBS, fixed with 4% formaldehyde, and treated with Alizarin red S (VWR International, Barcelona, Spain) at 2%, to stain the nodules of calcium formed in each of the samples, for 30 min. Subsequently, the samples were then washed with milliQ water twice and photographs were taken of all the wells using an inverted microscope (Nikon Eclipse TE2000-U, Nikon Corporation, Tokyo, Japan). For quantitative analysis of the staining, 200 μL of 10% acetic acid was added to each well and incubated for 30 min with constant agitation. The solution was then heated to 85 °C for 10 min, allowed to cool on ice, and centrifuged at 20,000× *g* for 15 min. The supernatant was transferred to a new tube, and 10% ammonium hydroxide was added to adjust the pH to 4.2. Subsequently, fifty microliters of each sample were added to a 96-well plate, and the absorbance was measured using a microplate reader (FLUOstar Omega, BMG Lab. Technologies, Cary, NC, USA). All experiments were performed in triplicate.

### 2.10. Statistical Analysis

Statistical analysis was performed using the analysis of variance (ANOVA) and Tukey’s multiple comparison tests using GraphPad Prism v7.0 (GraphPad Software Inc., San Diego, CA, USA). A *p*-value of <0.05 was considered statistically significant, corresponding to a 95% confidence interval.

## 3. Results

### 3.1. Effects on hDPSC Viability/Proliferation-MTT Assay

**Figure 2:**
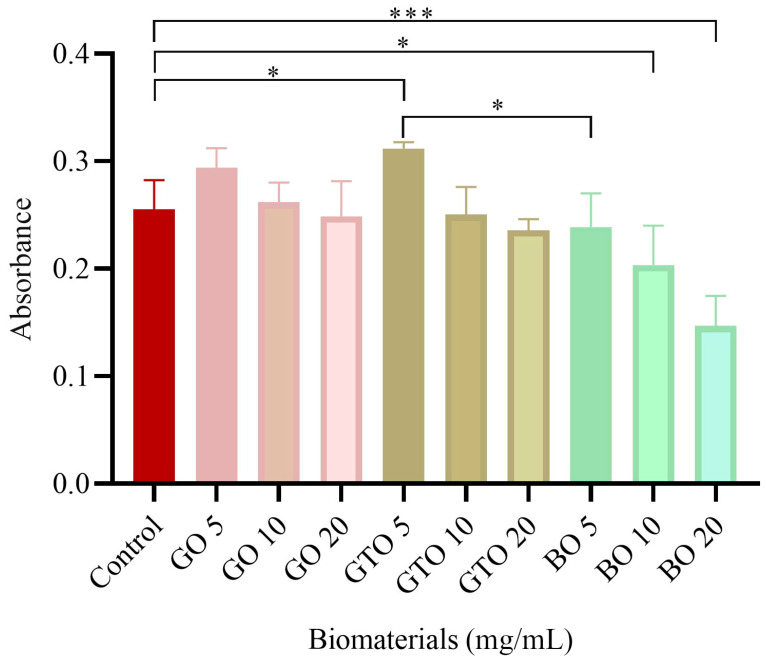
Cytotoxicity test results upon human dental pulp stem cell (hDPSC) viability after 72 h of culture of the biomaterials Osteobiol^®^ Gen-Os^®^ (GO), Osteobiol^®^ GTO^®^ (GTO) and Geistlich Bio-Oss^®^ (BO) for establishing their optimum concentration (5 mg/mL). Results expressed as the mean ± SD. * *p* < 0.05, *** *p* < 0.001.

The MTT assay was used to quantify the metabolic activity of the cells, as well as their proliferation, viability, and toxicity, in response to the biomaterials GO, GTO, and BO. The results of this assay (Figure 2) demonstrated that GTO at a concentration of 5 mg/mL enhanced viability as compared to the control (*p* < 0.05), while GO and BO did not show significant differences at the same concentration. However, at concentrations of 10 and 20 mg/mL, a significant decrease in viability caused by BO (*p* < 0.05 and *p* < 0.001, respectively) was observed. In contrast, GO and GTO did not demonstrate statistically significant differences as compared to the control group. The 5 mg/mL concentration was determined to be the optimal concentration and was used in all subsequent assays. On the other hand, when comparing the results between biomaterials at the working concentration (5 mg/mL), we found that BO showed lower viability with a significant difference compared to GTO, while no differences were observed between GTO and GO or between GO and BO.

### 3.2. Effects on hDPSC Viability-Cell Staining Assay

**Figure 3:**
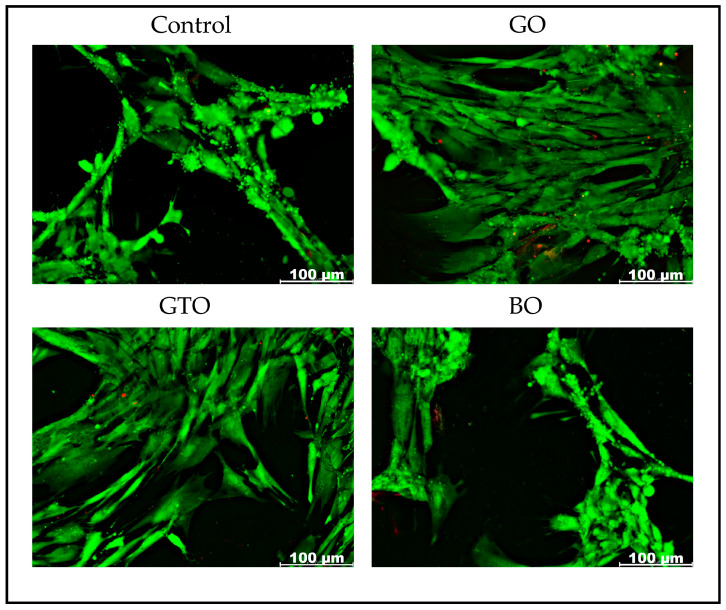
Fluorescence microscopy images of human dental pulp stem cells (hDPSCs) treated with the biomaterials Osteobiol^®^ Gen-Os^®^ (GO), Osteobiol^®^ GTO^®^ (GTO) and Geistlich Bio-Oss^®^ (BO) after 72 h of culture. Green staining indicates the presence of viable adherent cells, whereas red staining indicates cells with an impaired cell membrane.

Viability staining was conducted to qualitatively evaluate the response of hDPSCs to GO, GTO, and BO treatments after 72 h of incubation. Viable cells were identified by green fluorescence, while dead cells appeared red. The images obtained (Figure 3) revealed that the number of viable cells in GO and GTO was higher than that observed in the control group (untreated cells). In contrast, the BO cell count was found to be similar to that of the control group (Figure 3). Furthermore, the number of viable cells in both GTO and GO was superior to that observed in the BO treatment.

### 3.3. Effects on hDPSCs Cell Adhesion and Morphology by Scanning Electron Microscopy (SEM)

**Figure 4:**
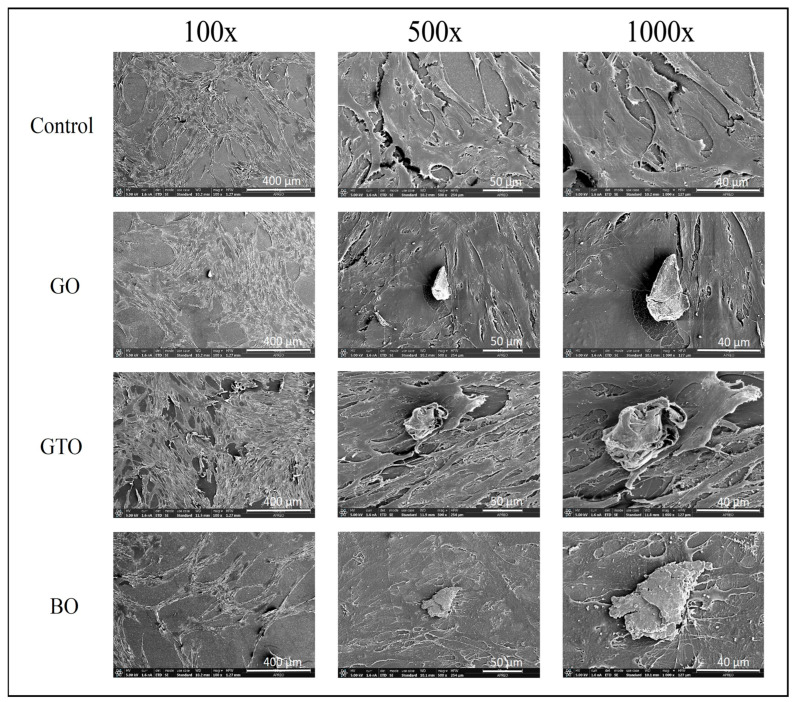
Scanning electron microscopy (SEM) micrographs of human dental pulp stem cells (hDPSCs) treated with the biomaterials Osteobiol^®^ Gen-Os^®^ (GO), Osteobiol^®^ GTO^®^ (GTO) and Geistlich Bio-Oss^®^ (BO) under ×100, ×500, and ×1000 magnification.

The interaction between the cells in contact with the biomaterials was assessed using scanning electron microscopy. As illustrated in Figure 4, SEM images obtained after 72 h of hDPSCs culture at magnifications of ×100, ×500, and ×1000 revealed that the cells were in contact with all biomaterials, and the morphology of the hDPSCs was similar in all treatment groups. Cells in the GO and BO groups demonstrated good cell adherence and cellular confluence. However, the GTO group exhibited the strongest adhesion results, with cells demonstrating greater confluence and adhesion to the surface of the biomaterial.

### 3.4. Effects on Cell Migration-Wound Heal Assay

**Figure 5:**
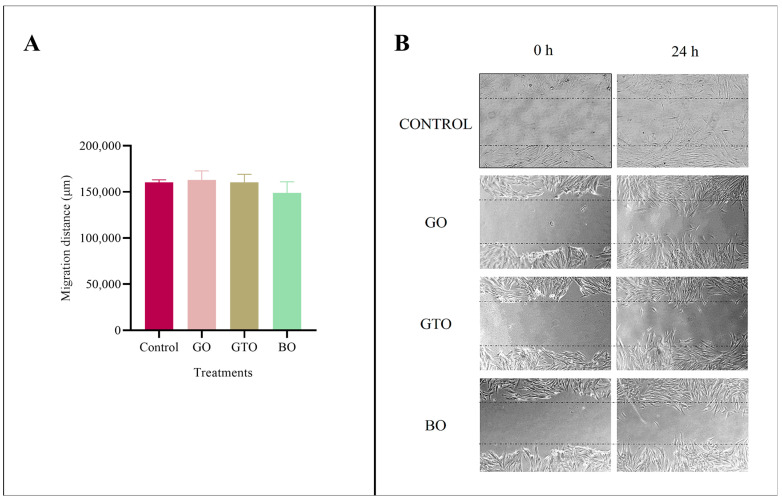
(**A**) Effects of the biomaterials Osteobiol^®^ Gen-Os^®^ (GO), Osteobiol^®^ GTO^®^ (GTO) and Geistlich Bio-Oss^®^ (BO) upon the migration of human dental pulp stem cells (hDPSCs) after 24 h of culture. The results are presented as mean ± standard deviation (**B**) Images of the effect of GO, GTO and BO on the migration ability of DPSCs after 24 h of culture.

The migration ability of hDPSCs treated with GO, GTO, and BO was assessed using the wound healing assay after 24 h of culture. The results of this assay (Figure 5) revealed no statistically significant differences in cell migration among the biomaterials or when compared to the control (untreated cells in complete medium).

### 3.5. Real-Time RT-PCR Assays-Adhesion, Migration, Proliferation, and Angiogenic Gene Expression

The mRNA expression levels of genes associated with adhesion and migration (*FN*, *SDF-1*, *COL1A1*) and with angiogenesis and proliferation (*VEGF*, *FGF2*), were assessed using real-time PCR.

**Figure 6:**
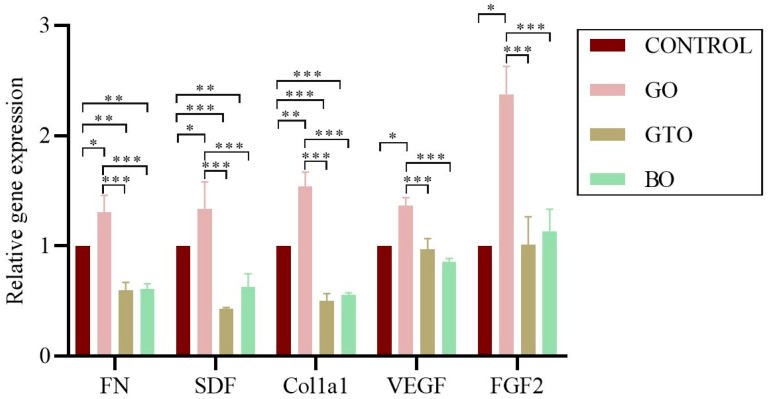
Effects of the biomaterials Osteobiol^®^ Gen-Os^®^ (GO), Osteobiol^®^ GTO^®^ (GTO) and Geistlich Bio-Oss^®^ (BO) on the expression of genes associated with adhesion and migration (*FN*, *SDF-1*, *COL1A1*) and angiogenesis and proliferation (*VEGF*, *FGF2*), in human dental pulp stem cells (hDPSCs) after 24 h of culture. Results are expressed as mean ± standard deviation: * *p* < 0.05, ** *p* < 0.01, *** *p* < 0.001.

The results revealed that GO significantly increased the expression of *FN*, *SDF-1*, *COL1A1*, *VEGF*, and *FGF2* genes as compared to the control group (*p* < 0.05, except for *COL1A1*, which was significant at *p* < 0.01). Furthermore, a significant decrease in *FN*, *SDF-1*, and *COL1A1* was observed in GTO and BO, while no significant differences were observed for *VEGF* and *FGF2* as compared to the control group. Likewise, direct comparisons showed that GO presented a greater expression of *FN*, *SDF-1*, *COL1A1*, *VEGF*, and *FGF2* compared to GTO and BO (*p* < 0.001), while no differences were observed between GTO and BO in the expression of these genes (Figure 6).

### 3.6. Real-Time RT-PCR Assays-Osteogenic Differentiation Gene Expression

**Figure 7:**
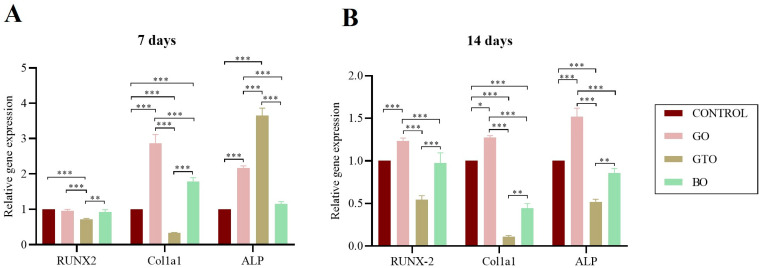
Effects of the biomaterials Osteobiol^®^ Gen-Os^®^ (GO), Osteobiol^®^ GTO^®^ (GTO) and Geistlich Bio-Oss^®^ (BO) on the expression of genes related to osteogenic differentiation (*RUNX2*, *COL1A1*, and *ALP*) of human dental pulp stem cells (hDPSCs) after (**A**) 7 days of culture and (**B**) 14 days of culture. Results are expressed as mean ± standard deviation * *p* < 0.05, ** *p* < 0.01, *** *p* < 0.001.

The expression of genes associated with osteogenic differentiation of hDPSCs exposed to GO, GTO, and BO was measured after 7 days (Figure 7A) and 14 days (Figure 7B) of culture, including *RUNX2* (a key osteogenic transcription factor), *ALP* (an early marker of osteoblastic activity), and *COL1A1* (a major component of the extracellular matrix of bone).

The results after 7 days of culture showed that GO significantly increased the expression of *COL1A1* and *ALP* (*p* < 0.001), while showing no significant differences in *RUNX2* as compared to the control group. However, at the 14-day interval, a significant upregulation of the three evaluated genes was observed in the GO-treated samples as compared to the control group (*RUNX2*, *ALP*: *p* < 0.001; *COL1A1*: *p* < 0.05).

On the other hand, with GTO, after 7 days of culture, a significant decrease was found in the expression of *RUNX2* and *COL1A1* as compared to the control (*p* < 0.001). However, a significant increase in the expression of *ALP* (*p* < 0.001) was also observed. Furthermore, after 14 days of culture, a significant decrease in the expression of all genes evaluated was observed as compared to the control (*p* < 0.001).

Finally, the use of BO at 7 days of culture increased the expression of *COL1A1* with respect to the control (*p* < 0.001), although significant differences in the expression of *RUNX2* and *ALP* were not obtained. Meanwhile, at 14 days, *COL1A1* expression significantly decreased with respect to the control (*p* < 0.001), and at 7 days, no differences were observed with respect to the control in the expression of *RUNX2* and *ALP*.

In terms of the comparison between the biomaterials, at 7 days, GO showed a greater expression of *RUNX2* and *COL1A1* (*p* < 0.001) compared to GTO, and of *ALP* and *COL1A1* (*p* < 0.001) compared to BO. Likewise, GTO increased *ALP* expression compared to GO and BO (*p* < 0.001), while BO showed greater expression of *RUNX2* and *COL1A1* (*p* < 0.01) in relation to GTO.

Furthermore, after 14 days, we observed that GO increased the expression of all evaluated genes relative to GTO and BO (*p* < 0.001), while BO showed greater expression of all genes compared to GTO (*RUNX2*: *p* < 0.001; *ALP* and *COL1A1*: *p* < 0.01).

### 3.7. Effects on In Vitro Biomineralization-Alizarin Red S Staining

**Figure 8:**
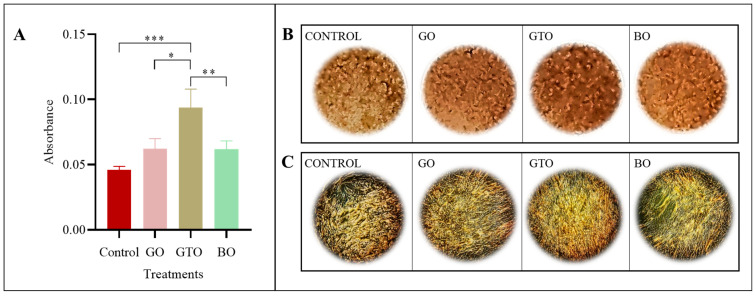
Effects of the biomaterials Osteobiol^®^ Gen-Os^®^ (GO), Osteobiol^®^ GTO^®^ (GTO) and Geistlich Bio-Oss^®^ (BO) on the mineralization of human dental pulp stem cells (hDPSCs) after 21 days of culture. (**A**) Mineralization assay results after 21 days of culture. Results expressed as the mean ± SD. * *p* < 0.05, ** *p* < 0.01, *** *p* < 0.001. (**B**) Alizarin red S staining in each treatment. (**C**) Images of the calcium deposits stained with Alizarin red S in each treatment.

The mineralization of hDPSCs treated with GO, GTO, and BO was quantitatively and qualitatively assessed after 21 days of culture (Figure 8). For this, we used Alizarin Red S staining that enabled us to visualize calcium nodules formed in each of the samples. The results of this assay (Figure 8A) showed that GTO increased mineralization, with a statistically significant difference as compared to the control (untreated cells in osteogenic medium) (*p* < 0.001) and also in relation to BO (*p* < 0.01) and GO (*p* < 0.05). In contrast, GO and BO exhibited no statistically significant differences. In addition, a comparative analysis of the staining images (Figure 8B,C) revealed that GTO had a significantly higher concentration of Alizarin Red-stained mineral deposits as compared to the control group as well as to GO and BO, while GO and BO had comparable staining levels to the control.

## 4. Discussion

This study evaluated the influence of collagen on the response of hDPSCs to three different bone regeneration scaffolds. The biomaterials analyzed exhibited notable differences in their composition, GO and GTO are of organic origin with distinct collagen formulations, whereas BO is an organic biomaterial lacking collagen [7,10]. To this end, a series of assays were employed, including viability assays, adhesion evaluation by scanning electron microscopy (SEM), a wound healing assay, RT-qPCR of genes associated with adhesion and migration (*FN*, *SDF-1*, *COL1A1*), associated with angiogenesis and cell proliferation (*VEGF*, *FGF2*), and osteogenic expression (*RUNX-2*, *COL1A1*, *ALP*), and a mineralization analysis by Alizarin red staining. This multifaceted approach enables us to ascertain the bioactivity of the biomaterials and to investigate the potential molecular mechanisms underlying their interaction with hDPSCs. As previously mentioned, collagen, as a primary structural component of the EMC, plays a fundamental role in mesenchymal cell adhesion and recruitment [21,22]. Our findings revealed a distinct response pattern for each material, suggesting that the composition and formulation of collagen may be influential factors in biocompatibility and regenerative potential.

Based on the MTT assay results, a concentration of 5 mg/mL of each biomaterial was selected as the working concentration for all subsequent experiments. This concentration demonstrated the best biocompatibility profile among the three materials evaluated, particularly for GTO, where a notable increase in cell viability was observed compared with both the control and the other biomaterials (BO and GO). In addition, it helped to avoid the potential cytotoxic effects observed at higher concentrations, as was the case with BO at 10 and 20 mg/mL. The selection of this concentration provided optimal conditions for accurately assessing cell migration, adhesion, and osteogenic differentiation without interference from biomaterial overload.

Furthermore, the MTT results were consistent with the images obtained from the viability staining assay, which showed a higher number of viable cells with the GO and GTO biomaterials in comparison to the control, while BO exhibited similar results to the control. Likewise, SEM images revealed that although the cells were in contact with all three biomaterials, GTO had the strongest interaction, as indicated by extensive cell spreading over its particles. This suggests a strong affinity and adhesive capacity, which could support subsequent processes such as migration and differentiation during later stages of culture [23,24].

The findings from these assays, particularly for GTO, can be attributed to the distinct physicochemical properties of the biomaterials evaluated. As previously mentioned, both GO and GTO contain collagen, although their compositions differ. GO is composed of collagenated cortico-cancellous granules, whereas GTO consists of 80% collagenated cortico-cancellous granules combined with 20% TSV gel enriched with collagen types I and III. A recent study corroborated these compositional differences by quantifying the collagen content in extracts of these materials, revealing that GTO contains four times more collagen than GO, while no collagen was detected in BO [7]. This variability likely has a significant impact on the cellular responses, as collagen plays a key role in processes such as cell adhesion, migration, differentiation, and mineralization [25,26,27].

The effects of the biomaterials in this study were evaluated using two complementary methods. First, the biomaterials were tested in an elution preparation for most of the assays. In contrast, for the SEM assay, the biomaterial particles were placed directly in the wells.

The elution approach allowed us to assess the influence of soluble factors, such as ions and collagen released by the biomaterials, on the activation of the cellular response.

In this context, the behavior of the evaluated biomaterials could be associated with their different degradation and release profiles, which are likely influenced by factors such as particle size and the content and formulation of collagen. For instance, the smaller particle size of GO and BO (250–1000 µm), compared to GTO (600–1000 µm), provides a greater surface area, which may have favored a faster release of their components [28,29]. A higher release of these components could affect the local microenvironment balance and therefore limit an optimal cellular response [30,31]. This effect could explain the potentially cytotoxic response observed in BO at higher concentrations, whereas the presence of collagen in GO appears to have mitigated such an outcome, resulting in a more favorable biocompatibility profile. In contrast, the elevated collagen content of GTO, attributable to its thermosensitive gel enriched with collagen types I and III, appears to have facilitated a more gradual and sustained release of bioactive components, thereby promoting stronger cellular interactions.

Likewise, the porosity of the biomaterials could also have modulated the extent of interaction between cells and biomaterials, as the interconnected spaces facilitate cell adhesion [32]. Our SEM analysis supports this, showing that all three biomaterials were biocompatible in this regard; however, GTO exhibited stronger adhesion, with cells actively extending over the biomaterial granules. This suggests that, although porosity is a key factor, the incorporation of a thermosensitive collagen gel in GTO may enhance cell–material interactions, thereby promoting optimal cell adhesion at early stages.

Furthermore, when migration was assessed using the wound healing assay, no significant differences were observed between the treatments and the control after 24 h of culture. These findings contrast with those from a previous study in human periodontal ligament (hPDL) cells, in which GO, GTO, and BO significantly increased cell migration after 24 h. This difference can be attributed to differences in both the cell lines used and the methodology employed [7].

However, despite the lack of functional differences in migration, relevant variations were observed in the expression of genes associated with cell migration and adhesion, such as *FN*, *COL1A1*, and *SDF-1*, as well as proangiogenic and proliferative genes such as *VEGF* and *FGF2*, across the different treatments. These molecular differences suggest an early activation of pathways involved in cell motility and interaction with the ECM, potentially preceding detectable morphological changes. As previously described, cell migration is a complex, sequential process that begins with gene activation signals, followed by cytoskeletal remodeling, dynamic adhesion to the ECM, and, ultimately, cell displacement [33].

Our findings indicate that the GO treatment enhances the expression of *FN*, *COL1A1*, and *SDF-1*, suggesting that this biomaterial could induce the early activation of the migration process, which may lead to more efficient cell migration and tissue remodeling processes in subsequent stages of the culture.

In contrast, GTO exhibited a reduced expression of *FN*, *SDF-1*, and *COL1A1* as compared to the control, while no significant differences were observed in the expression of *VEGF* and *FGF2*. The outcomes of this study may be interpreted not as a deficiency in biological activity, but rather as a consequence of an environment that is conducive to cell adhesion and anchorage, thereby obviating the need for the expression of these genes. It has been noted by several authors that an ECM microenvironment enriched with natural components, such as native collagen, can effectively interact with various cellular elements thanks to its triple helix structure. This interaction enables the ECM to modulate the effect of molecules involved in cell behavior through specific signaling pathways. Furthermore, variations in collagen density and composition, as well as the general culture context, have been demonstrated to influence growth factor signaling, cause phenotypic changes, and modify gene expression [34,35].

Consequently, the findings obtained with GTO imply that its inherent collagen content fosters a bioactive microenvironment, thereby facilitating efficient cell adhesion from the early stages. This phenomenon subsequently reduces the need for a heightened transcriptional response of genes associated with ECM synthesis. The validity of this hypothesis is substantiated by the findings of both cell viability assays and SEM analysis, which demonstrated a close interaction between cells and the biomaterial.

On the other hand, in the case of BO, a decrease in the expression of *FN*, *SDF-1*, and *COL1A1* was observed, while *VEGF* and *FGF2* expression did not significantly differ from the control. However, considering the previous functional assay results, these findings suggest that this biomaterial induces a limited activation of the molecular pathways involved in cell-ECM interaction, likely due to its predominantly anorganic nature.

Regarding the expression of proangiogenic and proliferative genes, as previously mentioned, GO was the only treatment that showed a significant increase in *VEGF* and *FGF2* as compared with the control and the other biomaterials, suggesting the early activation of angiogenesis-related pathways. In contrast, neither GTO nor BO showed differences in the expression of these genes, nor between each other. These findings contrast with those from a recent study evaluating protein secretion of these factors in hPDL cells treated with GO, GTO, and BO, where all treatments showed an increased secretion, especially GTO [7]. This discrepancy may be due to the complementary, but not always parallel, nature of gene expression and protein production, as processes such as post-translational regulation, release timing, and ECM interaction, can significantly influence the observed cellular behavior [36]. In this context, and considering the results of that study, our findings suggest that the angiogenic effects of the biomaterials, particularly GTO, may become more apparent at later stages or at a functional level, rather than during an early transcriptional phase.

The evaluated biomaterials also exhibited contrasting behaviors in terms of gene expression related to osteogenic differentiation and mineralization, likely due to differences in composition and bioactivity. Osteogenic differentiation is a dynamic process regulated by the sequential activation of transcription genes such as *RUNX2*, structural genes such as *COL1A1*, and enzymes such as ALP, marking different stages of osteoblast maturation from initial commitment to ECM mineralization. In this study, the expression of these genes, along with the mineralization results, revealed distinct responses between the biomaterials analyzed.

GO showed a progressive pattern in osteogenic gene activation. At day 7, a significant increase in *COL1A1* and *ALP* was observed as compared to the control, and at day 14, *RUNX2* expression increased, suggesting the sequential induction of the osteoblastic differentiation process. This profile is consistent with an active biological response, possibly favored by its preserved collagen content, which can act as a biochemical signal for mesenchymal cells, stimulating their osteoblastic commitment [26]. Although mineralization observed at 21 days was comparable to the control group, the early activation of osteogenic genes suggests that GO could induce a more effective response over prolonged culture periods.

In contrast, GTO showed an atypical pattern, with a lower expression of *RUNX2* and *COL1A1* at day 7, accompanied by a marked increase in *ALP*, followed by a significant reduction in this enzyme at day 14. However, GTO was the only biomaterial that showed a significantly greater Alizarin Red staining than the control at day 21, as well as an increase compared with BO and GO, indicating more efficient mineralization. This behavior, marked by an elevated early *ALP* activity followed by a decline, along with intense mineralization, suggests an accelerated progression toward late osteoblastic differentiation stages. This pattern has previously been associated with effective osteogenic differentiation, where early *ALP* activation reflecting osteoid matrix formation and its subsequent decline, along with increased mineral deposits, marks the transition to the mineralizing phase [35].

These findings suggest that GTO, due to its composition, may act as a bioactive matrix capable of modulating osteogenic differentiation through structural rather than transcriptional signals. This aligns with research showing that topographical surface cues can influence cytoskeletal organization, cell and nuclear morphology, and consequently gene expression [37,38,39,40,41].

Our findings with GTO and GO are consistent with the results reported by Di Tinco et al. [42], who demonstrated that both biomaterials exhibited good biocompatibility in terms of morphology and adhesion of hDPSCs. Despite methodological differences between the two studies, such as the data collection time points and the concentration used (10 mg/mL in their case), the results demonstrate points of convergence. In their study, cells exhibited an initial detachment after their first contact with the biomaterials; however, within 24 h, these cells regained adhesion and adopted a fusiform morphology. This finding aligns with the observations made at 72 h, where GTO demonstrated robust adhesion properties. This corresponds to our findings at 72 h, where GTO exhibited strong adhesion. Furthermore, the authors observed that GTO increased ALP activity after 10 days of culture, confirming its osteogenic potential, which is in line with our results.

On the other hand, BO exhibited a more conservative osteogenic profile. At day 7, an increase in *COL1A1* expression was observed, with no significant changes in *RUNX2* or *ALP*; and at day 14, *COL1A1* expression decreased, with no variation in the other genes evaluated. However, mineralization observed at day 21 was comparable to the control group, suggesting that although BO does not specifically activate osteogenic differentiation pathways, it effectively serves an osteoconductive function, providing adequate structural support for cell growth.

The clinical efficacy of BO is well-documented in the field of bone regeneration [43,44]. However, the results obtained in the present study evidence a negative effect on cell viability when using high concentrations of this biomaterial. This highlights the importance of carefully adjusting its concentration so as not to compromise cellular response, as has also been reported in previous studies [45,46]. Furthermore, a recent study demonstrated that the combination of BO with a peptide derived from the α-calcitonin gene (α-CGRP) significantly increased cell proliferation, *ALP* activity, mineralization nodule formation, and the expression of key osteogenic genes such as *RUNX2* and osteocalcin, as compared to the use of BO alone [47]. This suggests that incorporating bioactive biomolecules could be an effective strategy to enhance its biological performance. In this regard, although BO exhibits a moderate osteoconductivity, its functionality could be significantly optimized by approaches that integrate bioactive signals, especially in clinical scenarios that require faster, more efficient, and predictable bone regeneration.

Despite the limitations of this research, including the absence of comparative studies and a controlled in vitro environment lacking clinical stimuli, the findings demonstrate how each biomaterial acts through distinct mechanisms. GO exhibited the significant activation of genes associated with migration and osteogenic differentiation, especially in the initial stages, indicating its capacity to establish a favorable cellular environment conducive to bone regeneration. In contrast, GTO promoted a positive cellular response in all functional assays, demonstrating a high viability, an excellent cell coverage, and an efficient mineralization despite a lower gene activation. This behavior could be interpreted as the result of a bioactive environment provided by GTO, which may compensate for the need of a high endogenous expression of structural genes. In contrast, BO showed a more neutral molecular profile consistent with its osteoconductive role of acting as a structural scaffold without actively inducing osteoblastic differentiation.

These results support the applicability of biomaterials combined with dental pulp stem cells as a promising strategy for periodontal regeneration, and highlight their potential for clinical translation, where they could significantly improve the therapeutic options available for the repair of bone defects and dental supporting tissues differentiation. These findings emphasize the importance of considering both the composition of the biomaterials and the timing of their effects on cells, as cellular responses are not always immediately reflected at the morphological or transcriptional level. Consequently, a thorough evaluation encompassing the various stages of the differentiation process is imperative to ascertain the osteogenic potential of these biomaterials [48].

## 5. Conclusions

The biomaterials evaluated in this study, GO, GTO, and BO, demonstrated differential behaviors in their interactions with mesenchymal stem cell cultures, reflecting the importance of their composition in modulating cellular responses. Among them, GTO exhibited a distinctive profile, attributed to its higher collagen content and the incorporation of a thermosensitive collagen gel, which appears to create a bioactive microenvironment that promotes efficient mineralization even without strong transcriptional activation.

Overall, collagen-based xenografts demonstrate favorable interactions with hDPSCs, promoting osteogenic differentiation and mineralization. These findings support their clinical application in regenerative periodontal therapies, including guided bone regeneration and treatment of intraosseous defects.

## Figures and Tables

**Table 1:** Biomaterials.

Biomaterial/Batch	Manufacturer	Type/ Mechanism of Bone Formation	Origin/ Composition	Clinical Use
OsteoBiol^®^ GTO^®^ (GTO) 600–1000 µm/240129	Tecnoss^®^, Giaveno, Italy	Xenograft/ Osteoconductive	Porcine/80% Cortico-cancellous bone granules combined with 20% OsteoBiol^®^ TSV gel	Adaptable to any bone defect, horizontal augmentation procedures, and socket preservation
OsteoBiol^®^ Gen-Os^®^ (GO) 250–1000 μm/240146	Tecnoss^®^, Giaveno, Italy	Xenograft/ Osteoconductive	Collagenated cortico-cancellous bone mix with tissue collagen preserved	Alveolar ridge preservation, lateral access maxillary sinus lift, dehiscence regeneration, periodontal regeneration.
Geistlich Bio-Oss^®^ (BO) 0.25 mm–1 mm/82400382	Geistlich Pharma AG, Wolhusen, Switzerland	Xenograft/ Osteoconductive	Bovine/Deproteinized cancellous bone	Sinus lifts, periodontal defects, and long-term preservation of graft volume.

**Table 2:** Primer Sequence of genes used in qRT-PCR.

Assays	Gene	Primer Sequences (Forward/Reverse)
Migration and adhesion	Human Fibronectin (*FN*)	5′-TCCTTGCTGGTATCATGGCAG-3′/5′-AGACCCAGGCTTCTCATACTTGA-3′
	Collagen type 1 (*COL1A1*)	5′-CTACCTCCACCATGCCAAGT-3′/5′-GCAGTAGCTGCGCTGATAGA-3′
	Human Stromal cell-derived factor-1 (*SDF-1*)	5′-CGTGCTATGAAGGAAGATGGA-3′/5′-TGCCCAGTTCGTTTCAGT-3′
Angiogenesis and proliferation	Human Vascular endothelial growth factor (*VEGF*)	5′-GTCAGCCTGAGCTACAGATGC-3′/5′-CACTTTAGCTTCGGGTCAATG-3′
	Human Fibroblast growth factor 2 (*FGF2*)	5′-CGATGGATTCCAGTTCGAGTATG-3′/5′-TGTTCTTGCAGTGGTAGGTGATG-3′
Osteogenic differentiation	Alkaline phosphatase (*ALP*)	5′-TCAGAAGCTCAACACCAACG-3′/5′-TTGTACGTCTTGGAGAGGGC-3′
	Collagen type 1 (*COL1A1*)	5′-CGATGGATTCCAGTTCGAGTATG-3′/5′-TGTTCTTGCAGTGGTAGGTGATG-3′
	Runt-related transcription factor 2 (*RUNX2*)	5′-TCCACACCATTAGGGACCATC-3′/5′-TGCTAATGCTTCGTGTTTCCA-3′
Endogenous Gene	Human GAPDH (*GAPDH*)	5′-CCTGCACCACCAACTGCTTA-3′/5′-GGCCATCCACAGTCTTCTGAG-3′

## Data Availability

The original contributions presented in this study are included in the article. Further inquiries can be directed to the corresponding author.
